# Evaluation of Antimalarial Potential of Extracts from *Alstonia boonei* and *Carica papaya* in *Plasmodium berghei*-Infected Mice

**DOI:** 10.1155/2021/2599191

**Published:** 2021-10-06

**Authors:** Francis O. Atanu, Favour M. Idih, Charles O. Nwonuma, Helal F. Hetta, Salman Alamery, Gaber El-Saber Batiha

**Affiliations:** ^1^Department of Biochemistry, Faculty of Natural Sciences, Kogi State University, P.M.B. 1008, Anyigba, Nigeria; ^2^Department of Biochemistry, Faculty of Pure and Applied Sciences, Landmark University, Omu-Aran, Kwara State, Nigeria; ^3^Department of Medical Microbiology and Immunology, Faculty of Medicine, Assiut University, Assiut 71515, Egypt; ^4^Department of Internal Medicine, University of Cincinnati College of Medicine, Cincinnati, OH 45267-0595, USA; ^5^Department of Biochemistry, College of Science, King Saud University, PO Box 22452, Riyadh 11451, Saudi Arabia; ^6^Department of Pharmacology and Therapeutics, Faculty of Veterinary Medicine, Damanhour University, Damanhour 22511, Albeheira, Egypt

## Abstract

Extracts of *Alstonia boonei* and *Carica papaya* are used in herbal medicine for the treatment of malaria. This work investigated the phytochemical, antioxidant, and antimalarial effects of hydromethanolic extracts of *Alstonia boonei* and *Carica papaya*. A four-day chemosuppressive test was conducted to assess the ability of the extracts to prevent establishment of infection. Three doses of the extracts were administered—100, 200, and 400 mg/kg bw—prior to *Plasmodium berghei* challenge. Change in body weight, parasitemia, packed cell volume (PCV), and mean survival time was determined. A three-day curative test was also carried out on *Plasmodium berghei*-infected mice to determine the effects of the plant extracts (200 mg/kg bw) on parasitemia and biochemical indices of liver and kidney functions, lipid metabolism, and oxidative stress. The study revealed that the extracts possessed phenolic compounds (34.13 ± 1.90 mg GAE/g for *Alstonia boonei* and 27.99 ± 1.46 mg GAE/g for *Carica papaya*) and flavonoids (19.47 ± 1.89 mg QE/g for *Alstonia boonei* and 18.24 ± 1.36 mg QE/g for *Carica papaya*). *In vitro* antioxidant activity measured as total antioxidant power, total reducing power, and DPPH radical scavenging activity showed that the extracts possessed higher antioxidant activity than the reference compounds. The outcome of the chemosuppressive test revealed that whereas *Plasmodium berghei*-infected mice had high parasitemia, decreased mean survival time, exhibited loss of weight, and had low PCV, treatment with the extracts reversed the effects in a concentration-dependent manner. Similarly, the curative test revealed that the extracts significantly suppressed parasitemia compared with the malaria negative control group. This was mirrored by reversal of indices of hepatic toxicity (AST, ALT, and ALP levels), nephropathy (urea and creatinine levels), oxidative stress (SOD, CAT, GPx, GSH, and lipid peroxides), and dyslipidemia (TC, HDL, and TG levels and HMG-CoA reductase activity) in infected but treated mice compared with negative control. Put together, the results of this study demonstrate that the extracts of *Alstonia boonei* and *Carica papaya* possess antimalarial properties and are able to ameliorate metabolic dysregulations that characterize *Plasmodium berghei* infection. The phytoconstituents in these extracts are believed to be responsible for the pharmacological activity reported in this study.

## 1. Introduction

Malaria still remains a threat to life especially in children below the age of 5 in sub-Saharan Africa. According to the latest world malaria report, over 400,000 deaths were reported from over 200 million infections in 2019. The report also shows that the gains in the fight against malaria seem to have plateaued in recent times [1]. This is partly due to resistance to current frontline antimalarial drugs involving artemisinin-based combination therapy and the fact that the recently licensed malaria vaccine exhibits limited efficacy. The emergence of SARS-CoV-2 causing COVID-19 infections has further aggravated the scourge of malaria, limiting access to malaria health care services in the WHO African region due to travel restrictions. The fact that people with febrile illness are asked to stay at home as a preventive measure against the spread of SARS-CoV-2 also limits access to malaria treatment and could lead to a rise in malaria deaths in the nearest future if not properly managed. In the light of this, there is needed to look for potent alternatives of treating malaria in sub-Saharan Africa where the global burden of the disease is highest.

Medicinal plants have been used for a very long time to treat diseases, and they continue to be a pool for the discovery of drug leads [2]. In fact, current frontline antimalarial drugs are either derived directly from plants or are synthetically produced from a plant-derived chemical compound as template. For example, artemisinin was isolated from *Artemisia annua* [3], while quinine was isolated from *Cinchona officinalis* [4]. These two compounds form the basis for artemisinin-based combination therapy and the synthesis of chloroquine (or hydroxychloroquine), respectively. According to a recent review of plant-derived drugs and their contribution to the global disease pandemic, it is believed that the future of drug discovery lies in plant-derived natural products [5].

In Nigeria, folklore use of medicinal plants is a common practice due to accessibility, cultural acceptability, and relative affordability. However, this is usually at the risk of safety as most of the traditional remedies are without scientifically proven efficacy. Besides, the dosage of administration most of the time is arbitrary, increasing the risk of side effects and toxicity. One common practice among herbalists in Nigeria is the fact that these traditional remedies are derived from extraction using hydrophilic solvents such as water and ethanol. Some of the most common plants from which extracts are used as traditional remedies for diseases include *Alstonia boonei*, *Carica papaya*, *Vitex doniana*, *Tapinanthus dodoneifolius*, *Senna alata*, and *Terminalia catappa*. It is therefore expedient to study these fractions scientifically toward discovery of drug leads.

*Alstonia boonei* is a deciduous tree belonging to the family Apocynaceae. It can grow as high as 40 m and up to 27 m without branches [6]. Extracts from the plant have demonstrated antimalarial activities and management of anemia, which is characteristic of malaria infection. Its antimalarial activities have been examined using animal models both singly or in combination with other plants. For instance, Idowu et al. [7] showed that combination of *Alstonia boonei* with *Picralima nitida*, and *Gongronema latifolium*, had antiplasmodial activity with no evidence of toxicity to the liver or kidneys. A more recent study by Omoya and Oyebola [8], revealed that the leaves of *Alstonia boonei* had higher chemosuppressive activity than the stem extract. Extracts of *Alstonia boonei* have been reported to be rich in phytochemicals, which could target a plethora of plasmodium metabolic pathways.

One of such reports showed that a compound isolated from *A*. *boonei* inhibits the activity of both lactate dehydrogenase and plasmepsin II in malaria parasites [9]. Stem bark infusion of the plant has shown anticancer properties against colon cancer cell line [10] as well as antiulcerative properties [11]. Aqueous extracts of the leaf of this plant has also demonstrated anti-inflammatory and antioxidant activities in experimental rats [12].

*Carica papaya* is a plant commonly referred to as papaya or pawpaw. The fruit of the plant is eaten globally, while the leaves are used in some parts of the world to treat human diseases such as malaria, typhoid, piles, and diabetes. Literature search reveals scientific evidence for the antimalarial activity of *C*. *papaya* extracts when administered singly or in combination with other plant extract or with approved drugs [13–16]. *C*. *papaya* is rich in bioactive phytochemicals of diverse families. Glycosides of flavanols and caffeoyl derivatives obtained from the decoction of *C*. *papaya* leaves have shown synergistic potency with artesunate against *P*. *falciparum* and *P*. *berghei*, preventing parasite recrudescence [17].

However, despite the above reports, there are no available data on the antiplasmodial effect of aqueous and ethanolic extracts of the leaf of these plants to justify their use in traditional medicine for the treatment of malaria. Therefore, this study seeks to provide scientific evidence for the use of aqueous and ethanolic extracts from the leaves of these plants locally in traditional medicine. Compounds identified from the phytochemical screen can be investigated further for the discovery of potent drug leads against malaria.

## 2. Materials and Methods

### 2.1. Plant Collection, Identification, and Processing

Leaves of *Alstonia boonei* and *Carica papaya* were collected from Kogi State University, Anyigba, Nigeria campus, and identified in the Department of Botany by Prof. S.S. Usman. The leaves were washed with water, dried to constant weight at room temperature, and pulverized using an electric blender. The pulverized plant material was extracted in 50% methanol (H_2_O/ethanol) for 48 hours followed by filtration and concentrating of the filtrate using a rotary evaporator. The concentrated extract was allowed to evaporate to dryness. The plant extracts were stored at 4°C.

### 2.2. Phytochemical Screening of Plant Extracts

#### 2.2.1. Qualitative Phytochemical Screening

Phytochemical screening of the plant extracts were carried out according to methods of Trease and Evans [18] and Harborne [19].

#### 2.2.2. Total Phenolics

The total phenolic of the extracts was determined using the Folin–Ciocalteu assay method of Singleton and Rossi [20]. The total phenolic content of the plants was then calculated and expressed as mg gallic acid equivalent (GAE)/g extract.

#### 2.2.3. Total Flavonoids

Total flavonoid content was determined using the aluminum chloride colorimetric method by Zhilen as described by Arogba [21]. The concentrations were expressed as mg quercetin equivalent (QUE)/g extract.

### 2.3. In Vitro Antioxidant Evaluation of Plant Extracts

#### 2.3.1. Total Antioxidant Power of Extracts

The total antioxidant power of the extracts was evaluated according to the method described by Sharifia et al. [22]. Briefly, concentrations ranging from 25 to 400 *µ*g/ml of the extracts were prepared in DMSO. 100 *µ*_l_ of each concentration of the extracts was mixed with 1 ml of the working reagent (0.6 M sulfuric acid, 28 mM sodium phosphate, and 4 mM ammonium molybdate). The reaction mixture was incubated at 95°C for 90 min and then cooled, and absorbance was taken at 695 nm against a blank.

Vitamin C was used as standard. The EC_50_ (effective concentration) of the extracts was extrapolated from a plot of absorbance versus concentrations of each extract. The concentration that gave 0.5 absorbance was taken as the EC_50_.

#### 2.3.2. Reducing Power of Extracts

The reducing power assay was performed according to the method described by Yen et al. [23]. Plant extract with concentrations ranging from 25 to 400 *µ*g/ml was mixed with 2.5 mL of 0.2 M phosphate buffer (pH 6.6) and 2.5 mL of potassium ferricyanide (1% w/v). The mixture was incubated at 50°C for 20 min. Then, 2.5 ml of 1% trichloroacetic acid was added to the mixture to stop the reaction followed by centrifugation at 3000 rpm for 10 min. The supernatant (2.5 mL) was mixed with distilled water (2.5 mL) and 0.1% FeCl_3_ (0.5 mL), and then absorbance was measured at 700 nm against a blank.

#### 2.3.3. DPPH Radical Scavenging Property of Extracts

The DPPH radical scavenging activity was evaluated according to the method of Blois [24]. IC_50_, which is the concentration at which 50% of the DPPH radical is scavenged, was determined from the curve of percentage inhibition plotted against various concentrations of the extracts. The results obtained were compared with the IC_50_ of quercetin.

### 2.4. In Vivo Experiments

#### 2.4.1. Experimental Animals and Malaria Parasite

Male albino mice obtained from the animal house of Ahmadu Bello University, Zaria, Nigeria, were used for this study. The mice were kept in metal cages and allowed to acclimatize for the period of 14 days before been infected with the malaria parasite. Animals were fed commercial rat chow and water *ad libitum* throughout the period of the experiments. Animal sacrifice was humanely carried out according to acceptable international standards.

*Plasmodium berghei* NK65 was a gift from the Department of Parasitology, National Institute for Medical Research, Yaba, Lagos, Nigeria. The parasite was maintained in a mice host by serial passage of infected mouse to uninfected naïve mouse. The level of parasitemia was monitored by microscopic examination of blood smears.

#### 2.4.2. Acute Toxicity Test

The acute toxicity test in both plants was carried out according to OECD no. 425 guideline as described by Habte et al. [25,26]. Male mice were fasted overnight and administered with a single oral dose of 2000 mg/kg body weight of hydroethanolic extracts of *Alstonia boonei* and *Carica papaya*. The mice were fasted for additional two hours before given food and water. The mice were thereafter observed for 24 hours. The mice were further observed for 14 days for signs of toxicity such as convulsion, aggression, loss of appetite, hair erection, and muscle tremor.

#### 2.4.3. Induction and Monitoring of Parasitemia

Chloroquine-sensitive *Plasmodium berghei* NK65 strain was maintained in mice by passage of blood from infected to healthy mouse once every 4–5 days. Induction of mouse was done by intraperitoneal injection of 200 *µ*l of blood (20–30% parasitemia) from an infected mouse (blood collected via cardiac puncture). Parasitemia was monitored by standard methods; thin blood smears were made on glass slides, fixed using ethanol, and stained using Giemsa stain, and parasitemia was counted using a microscope and was calculated as a percentage of infected red blood cells (RBCs) relative to the total number of cells in a microscopic field at ×100 magnification as given below:

(1) $$\begin{array}{c} Parasitemia\;\left(\%\right)=\frac{Total\;number\;of\;parasitise\;d\;RBCs}{Total\;number\;of\;RBCs}\times \;100. \end{array}$$

#### 2.4.4. Experiment I: Suppressive Test

Mice were infected by intraperitoneal injection of parasitized blood. Treatment of the respective groups commenced 3 h after infection. Treatments lasted 4 days. The percentage suppression of parasitemia was calculated as below:

(2) $$\begin{array}{c} Percent\;Suppression=\frac{Parasitemia\;in\;negative\;control-Parasitemia\;in\;treate\;d\;group}{Parasitemia\;in\;negative\;control}\times \;100. \end{array}$$

Mice were observed for 29 days post infection, and the mean survival time was calculated as follows:

(3) $$\begin{array}{c} Mean\;Survival\;Time\;\left(MST\right)=\frac{Sum\;of\;survival\;da\;ys\;of\;all\;mice\;in\;a\;group}{Total\;number\;of\;mice\;in\;the\;group}. \end{array}$$

#### 2.4.5. Experiment II: Curative Test

Mice were infected by intraperitoneal injection of parasitized blood. Treatments of the respective groups commenced 72 h after infection. From the results of the four-day suppressive test, a median daily dose of 200 mg/kg of the extracts was selected for the curative test. Treatments lasted 4 days.

#### 2.4.6. Animal Sacrifice and Collection of Blood Samples

At the end of the experiments, mice were anesthetized using chloroform in a glass jar and blood was collected via cardiac puncture. The blood was allowed to stand on the bench for 1 h to clot followed by centrifugation at 5,000 rpm for 10 min to separate serum.

### 2.5. Determination of Packed Cell Volume (PCV)

Packed cell volume (PCV) was determined using the capillary method. Tail blood was collected into a heparinized hematocrit tubes. The tubes were sealed with crystal seal and thereafter centrifuged for about 10,000 rpm for 5 minutes. The volume of cells was calculated according to the following formula:

(4) $$\begin{array}{c} PCV=\frac{Erythrocyte\;volume}{Total\;bloo\;d\;volume}\times 100. \end{array}$$

### 2.6. Biochemical Assays

Diagnostic kits were used for the analysis of serum indices for kidney function (urea, creatinine), liver function (AST, ALT, ALP), lipid profile (HDL, TG, total cholesterol), HMG-CoA reductase, and oxidative stress markers (lipid peroxidation, catalase, superoxide dismutase, glutathione peroxidase). Assays were performed according diagnostic kits' manufacturer's protocols.

### 2.7. Statistical Analysis

InStat GraphPad software was used for analysis of variance (ANOVA) to ascertain significant differences between means. Differences were considered statistically significant at *P* < 0.05.

## 3. Results and Discussion

Medicinal plants have been used for decades in the treatment of diseases, and they continue to be a pool for the discovery of drug leads [2]. The present study reports the antimalarial activity of *Alstonia boonei* and *Carica papaya* in *P*. *berghei*-infected mice. This study showed that the hydroethanolic extracts of *Alstonia boonei* and *Carica papaya* contain alkaloids, flavonoids, phenolics, tannins, saponins, anthraquinones, and terpenoids (Table 1). Alkaloids, flavonoids, and tannins are known for their antioxidant, antimalarial, antimicrobial, anticancer, and anti-inflammatory activities [27, 28]. The extracts had total phenolics (34.13 ± 1.90 mg GAE/g for *Alstonia boonei* and 27.99 ± 1.46 mg GAE/g for *Carica papaya*) and total flavonoid (19.47 ± 1.89 mg QE/g for *Alstonia boonei* and 18.24 ± 1.36 mg QE/g for *Carica papaya*). Phenolics have been reported to possess antioxidant, antimalarial, antimutagenic, anticancer, and anti-inflammatory capacities. The hydroxyl group substituent of aromatic benzene rings are responsible for this biological activity due to their capacity to eliminate or absorb free radicals and to chelate reactive oxygen species molecules. The potency of phenolics is proportional to the number of hydroxyl (OH) groups present in their aromatic rings [29]. It is believed that flavonoids exert their antimalarial activity by inhibiting fatty acid biosynthesis (FAS II) in the parasite [30,31]. Some flavonoids have also been reported to inhibit the influx of L-glutamine and myoinositol into infected erythrocytes [32].

**Figure 1:**
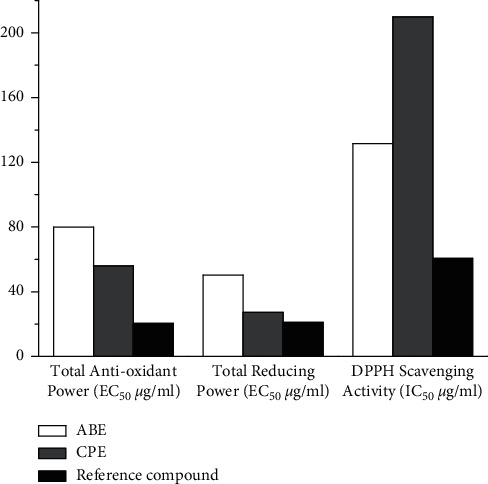
*In vitro* antioxidant screening of extracts of *Alstonia boonei* and *Carica papaya*.

Over the years, there have been reports validating the fact that plants do possess antioxidant activity though in varying degrees. In this study, it was observed (Figure 1) that the hydroethanolic extracts of *Alstonia boonei* and *Carica papaya* had substantial *in vitro* antioxidant activity almost comparable to ascorbic acid. This is not unconnected with the phytochemicals present in the extracts. The DPPH scavenging activity and TAC reported in this study also gave credence to the antioxidant activity of the extracts.

The extracts of *Alstonia boonei* and *Carica papaya* did not cause any death within the 24 hours of acute toxicity test. Furthermore, observation of the mice for 14 days did not reveal any behavioral changes characteristic of toxicity. This shows that the extract may be safe for *in vivo* administration up to 2000 mg/kg. Our result of acute toxicity test is similar to the results of the study by Enechi et al., which showed that *Alstonia boonei* was tolerated up to a dose of 5000 mg/kg [33]. Similarly, Solikhah et al. reported zero death for mice administered the leaf extract of *Carica papaya* up to a dose of 3000 mg/kg [34].

**Figure 2:**
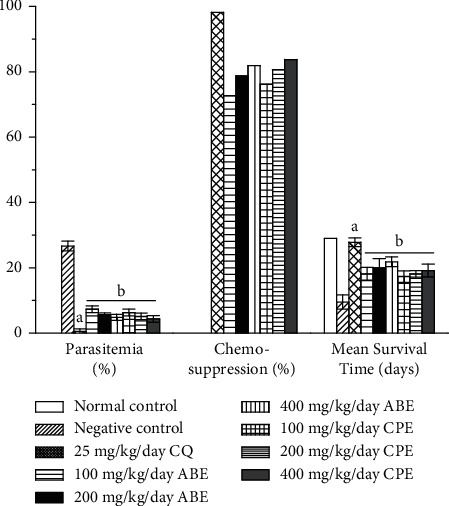
Chemosuppressive antimalarial effect of hydroethanolic extracts of *Alstonia boonei* and *Carica papaya* in *Plasmodium berghei*-infected mice. Values are presented as mean ± SD of six (6) determinations. *a* < 0.001 compared with negative control. *b* < 0.001 compared with CQ group.

**Figure 3:**
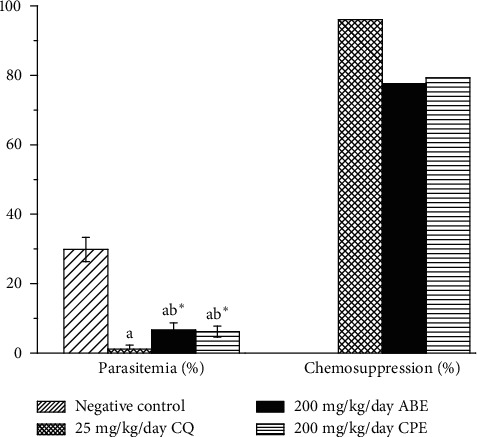
Curative antimalarial activity of hydroethanolic plant extracts of *Alstonia boonei* and *Carica papaya* in *Plasmodium berghei*-infected mice. Values are presented as mean ± SD of six (6) determinations. *a* < 0.001 compared with negative control group; *b∗* < 0.01 compared with CQ group.

**Figure 4:**
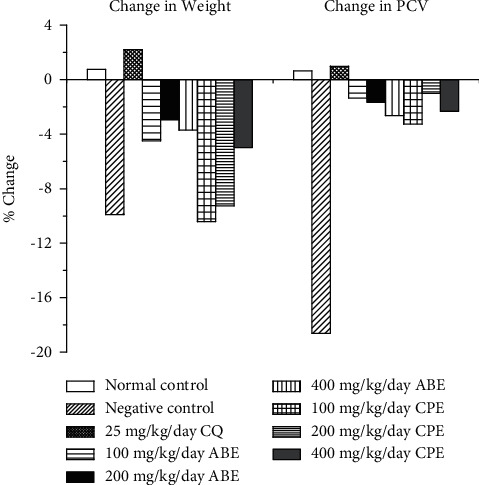
Changes in body weight and packed cell volume (PCV) in *Plasmodium berghei*-infected mice treated with chemosuppressive doses of hydroethanolic extracts of *Alstonia boonei* and *Carica papaya*.

Figures 2 and 3 show the chemosuppressive and curative antimalarial effects of the hydroethanolic extracts of *Alstonia boonei* and *Carica papaya* in *Plasmodium berghei*-infected mice. The extracts had a significant chemosuppressive and curative antimalarial effect on the infected mice when compared with the untreated group. This is believed to be as a result of some of the bioactive compounds such as flavonoids, phenolics, alkaloids, and anthraquinones present in the extracts [27–29]. The extracts reduced parasitemia and increased mean survival time in the treated group. It was also observed in this study (Figure 4) that though the extracts could not support increase in body weight and packed cell volume (PCV) while exerting their antimalarial effect, respectively, they were able to reduce the decreasing effect of the parasite on the body weight and PCV of the treated groups.

**Figure 5:**
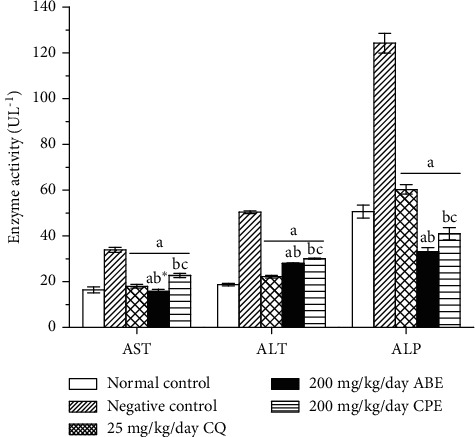
Liver function parameters of *Plasmodium berghei*-infected mice treated with hydroethanolic plant extracts of *Alstonia boonei* and *Carica papaya*. Values are presented as mean ± SD of six (6) determinations. *a* < 0.001 compared with negative control; *b* < 0.001, *b∗* < 0.01 compared with CQ group; *c* < 0.001 compared with 200 mg/kg/day ABE.

**Figure 6:**
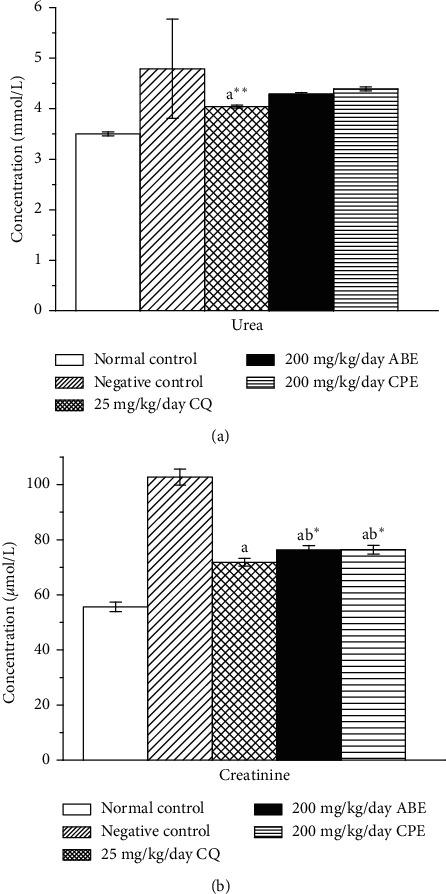
Kidney function parameters of *Plasmodium berghei*-infected mice treated with hydroethanolic plant extracts of *Alstonia boonei* and *Carica papaya*. Values are presented as mean ± SD of six (6) determinations. *a* < 0.001, *a∗∗* < 0.05 compared with negative control; *b∗* < 0.01 compared with CQ group.

Malaria is known to cause liver and kidney damage especially is severe cases. In fact, it is the first parasitic infection to be clearly associated with glomerular diseases in the tropical region [24]. Hepatic dysfunction and jaundice are common features of severe malaria [35–37]. The extracts decreased serum AST, ALT, and ALP in the treated groups when compared with the untreated group (Figure 5); this indicates hepatoprotection. The increase in the ALP in the untreated group may be due to compromised cell membranes integrity. Similarly, the increase in the AST and ALT levels could be due to leakage from the hepatocytes due to damage by the parasite [38,39]. Severe malaria can lead to glomeruli disease condition as well as tubules and interstitial region disorders. It was observed in this study that the extracts reduced the impact of the infection on the kidney. This is seen in the decrease of urea and creatinine (biomarkers for kidney function) in the treated groups compared with the negative untreated group (Figure 6). An alternative explanation for disturbances in urea and creatinine concentrations could be due to sequestration of the parasite into the renal microvasculature bed, which may lead to ischemia [40]. Liver and kidney diseases associated with malaria are basically as a result of erythrocyte abnormalities. Parasitized red cells tend to adhere to healthy erythrocytes, blood platelets, and capillary endothelium, and this in turn leads to formation of rosettes and clumps, which impair microcirculation [41].

**Figure 7:**
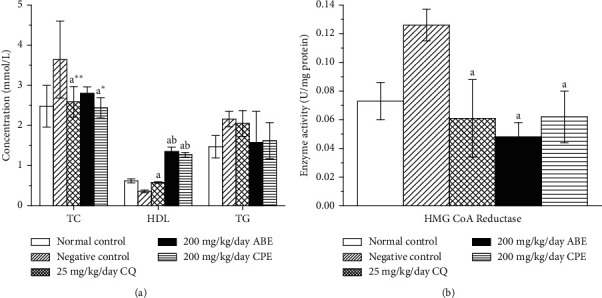
Lipid profile (a) and HMG-CoA reductase activity (b) of *Plasmodium berghei*-infected mice treated with hydroethanolic plant extracts of *Alstonia boonei* and *Carica papaya*. TC: total cholesterol, HDL: high-density lipoprotein, TG: triglycerides. Values are presented as mean ± SD of six (6) determinations. *a* < 0.001, *a∗* < 0.01, *a∗∗* < 0.05 compared with negative control; *b* < 0.001 compared with CQ group.

It has been hypothesized that malaria parasites use cholesterol and phospholipids from its host, resulting in a decrease of serum HDL-cholesterol [42]. This and other factors are responsible for dyslipidemia in *Plasmodium*-infected animals. The result from this study showed an increase in HDL-cholesterol of the groups treated with the extracts, while a decrease in HDL-cholesterol was observed in the untreated groups (Figure 7). The fast multiplication of intrahepatic *Plasmodium* necessitates a high demand of lipid for organelle biogenesis [43]. In a study where microarray-based approach to profile hepatocyte response to *Plasmodium* infection was utilized, the results revealed upregulation of genes coding for sterol synthesis and lipid metabolism. This evidently might be responsible for the increased activity of HMG-CoA reductase activity of the pathway of cholesterol synthesis. Previous studies also reported increased concentrations in TG and VLDL and simultaneously a decrease in HDL and LDL in malarial infection condition [43]. The increase in the HDL and decrease in TG reported in the study in the treated groups could be indicative of the therapeutic potential of the extracts.

**Figure 8:**
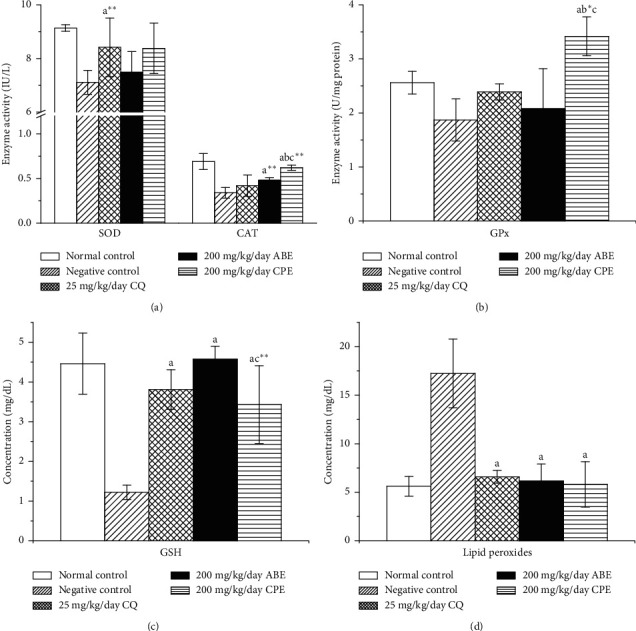
Oxidative stress markers of *Plasmodium berghei*-infected mice treated with hydroethanolic plant extracts of *Alstonia boonei* and *Carica papaya*. SOD: superoxide dismutase, CAT: catalase, GPx: glutathione peroxidase, GSH: glutathione. Values are presented as mean ± SD of six (6) determinations. *a* < 0.001, *a∗∗* < 0.05 compared with negative control; *b* < 0.001, *b∗* < 0.05 compared with CQ group; *c* < 0.001, *c∗∗* < 0.05 compared with 200 mg/kg/day ABE.

The extracts caused increased in antioxidant enzymes (SOD, catalase, GPX, and GSH) activity while decreasing lipid peroxides concentration in the treated groups; this is in contrast with the untreated group where decrease in antioxidant enzymes activity and increase in lipid peroxides were observed (Figure 8). This result correlates with what was observed in the *in vitro* antioxidant assay where the extracts were seen to possess substantial antioxidant activity. The increased enzyme activity is an indication of integrity or intactness of the enzyme, while the decreased activity in negative control group may be indicative of compromised enzyme integrity by the increased production of free radicals. The lipid peroxides level reduced while GSH levels increased significantly across the treatment groups. This result complements the ability of the extracts to increase the activity of antioxidant enzymes in parasitized mice. The decrease in peroxides and increase in GSH may be an indication of recovery from the metabolic anomalies elicited by *Plasmodium* infection.

Put together, phytochemicals present in hydroethanolic extracts of *Alstonia boonei* and *Carica papaya* demonstrated antimalarial activity in *Plasmodium berghei*-infected mice. The extracts reversed hepatic toxicity, nephropathy, oxidative stress, and dyslipidemia in infected mice.

## 4. Conclusions

The hydroethanolic extracts of *Alstonia boonei* and *Carica papaya* possess bioactive compounds responsible for the antioxidant and antimalarial activity reported in this study. Further studies should be directed toward identifying and characterizing these bioactive active compounds for proper utilization of their potentials in disease management.

## Figures and Tables

**Table 1:** Phytochemical profile and antioxidant activity of hydroethanolic extracts of *Alstonia boonei* and *Carica papaya*.

Parameter	*Alstonia boonei*	*Carica papaya*
Alkaloids	+	+
Flavonoid	+	+
Phenolics	+	+
Tannins	+	+
Terpenoids	+	+
Saponins	+	+
Steroids	+	+
Anthraquinones	+	+
Cardiac glycosides	+	+
Extraction yield (%)	12.53 ± 0.67	8.10 ± 0.56
Total phenolics (mg GAE/g)	34.13 ± 1.90	27.99 ± 1.46
Total flavonoid (mg QE/g)	19.47 ± 1.89	18.24 ± 1.36

## Data Availability

All data are included in the article.
